# The Molecular and Genetic Interactions between Obesity and Breast Cancer Risk

**DOI:** 10.3390/medicina59071338

**Published:** 2023-07-21

**Authors:** Ghada M. A. Ajabnoor

**Affiliations:** 1 Department of Clinical Biochemistry, Faculty of Medicine, King Abdulaziz University, Jeddah 21589, Saudi Arabia; gajabnour@kau.edu.sa; 2 Food, Nutrition and Lifestyle Unit, King Fahd Medical Research Centre, King Abdulaziz University, Jeddah 21551, Saudi Arabia; 3 Saudi Diabetes Research Group, Faculty of Medicine, King Abdulaziz University, Jeddah 21589, Saudi Arabia

**Keywords:** obesity, breast cancer, adipokines, insulin-like growth factors, phosphatidylinositol 3-phosphate, dyslipidemia, epigenetics, single nucleotide polymorphism

## Abstract

Breast cancer (BC) is considered the leading cause of death among females worldwide. Various risk factors contribute to BC development, such as age, genetics, reproductive factors, obesity, alcohol intake, and lifestyle. Obesity is considered to be a pandemic health problem globally, affecting millions of people worldwide. Obesity has been associated with a high risk of BC development. Determining the impact of obesity on BC development risk in women by demonstrating the molecular and genetic association in pre- and post-menopause females and risk to BC initiation is crucial in order to improve the diagnosis and prognosis of BC disease. In epidemiological studies, BC in premenopausal women was shown to be protective in a certain pattern. These altered effects between the two phases could be due to various physiological changes, such as estrogen/progesterone fluctuating levels. In addition, the relationship between BC risk and obesity is indicated by different molecular alterations as metabolic pathways and genetic mutation or epigenetic DNA changes supporting a strong connection between obesity and BC risk. However, these molecular and genetic alteration remain incompletely understood. The aim of this review is to highlight and elucidate the different molecular mechanisms and genetic changes occurring in obese women and their association with BC risk and development.

## 1. Introduction

Breast cancer (BC) is the second leading cause of death among women worldwide [1,2]. BC accounts for up to 25% of newly diagnosed cancer cases in women globally [1,3]. Around one-half of women with metastatic BC who undergo therapy will develop metastatic relapse within five years [4]. Recent epidemiological studies indicated that BC incidence is more than fourfold higher in developed than developing countries [5]. According to recent BC prevalence reports, BC is a leading cause of cancer death among women aged 20–59 years in the United States [6]. Additionally, patients with metastatic BC generally have a poor five-year survival rate of 25% [7,8,9]. In Saudi Arabia, BC was the most diagnosed type of cancer among women and the second leading cause of death after leukemia in 2018 [10]. While BC incidence is lower in Saudi Arabia compared to many Western countries [10], there is growing evidence that it is rapidly increasing in Saudi Arabia [10].

Various well-known risk factors are associated with BC risk, such as age, genetic family history, and reproductive factors [11,12,13]. Reproductive risk factors show stronger associations with BC risk in postmenopausal women [11]. However, 30% of BCs are associated with modifiable risk factors such as obesity, alcohol intake, and smoking [12,14]. Therefore, BC prevention and risk reduction can be achieved by lifestyle changes, which are crucial for improving the quality of life of women [15].

Epidemiological studies have shown obesity and weight gain to be associated with BC risk and incidence [3,16]. Obesity is a pandemic health problem globally, affecting millions worldwide [17,18]. It is well known as dysregulation in body metabolism that induces a low-grade inflammatory condition [17,19]. Various health complications and diseases are associated with obesity or high body mass index (BMI), including diabetes, cardiovascular diseases, and cancer. The World Health Organization reported that >600 million adults and 15% of women were obese worldwide in 2014 [20]. Many studies have also highlighted a significant association between high obesity prevalence and the pathogenesis of various types of cancers, including BC risk in pre- and post-menopausal females [21,22]. Moreover, obesity has been associated with poor BC prognosis, increasing risks off disease relapse about 35% to 40% and mortality [18,20,23]. Different studies indicated that morbid obesity increases the risk of death due to BC by about 2.26 fold [24]. Hence, obese BC patients are at high risk of different types of clinical complications of treatments, including breast surgery, radiotherapy, and systemic therapy [25]. Thus, obese patients undergoing surgery are at high risk for anesthesia complications, including difficulty in maintaining ventilator support and intubation, compared to non-obese BC patients [25]. Other surgical complications for obese or high BMI women with BC include wound dehiscence, infection, and lymphoedema development in pre- or post-operative cases [26]. In addition, in cases of breast reconstruction surgery post mastectomy for BC patients, there are increased risks of venous thromboembolism in high BMI females [27]. Other BC therapy, including systemic treatments such as chemotherapy, hormonal therapy, and radiotherapy in high BMI or obese women, showed poor outcomes of treatment and survival despite receiving a higher dose of treatment [25]. Therefore, the efficacy of treatment for obese BC is significantly low due to challenges of disease management [28].

**Figure 1:**
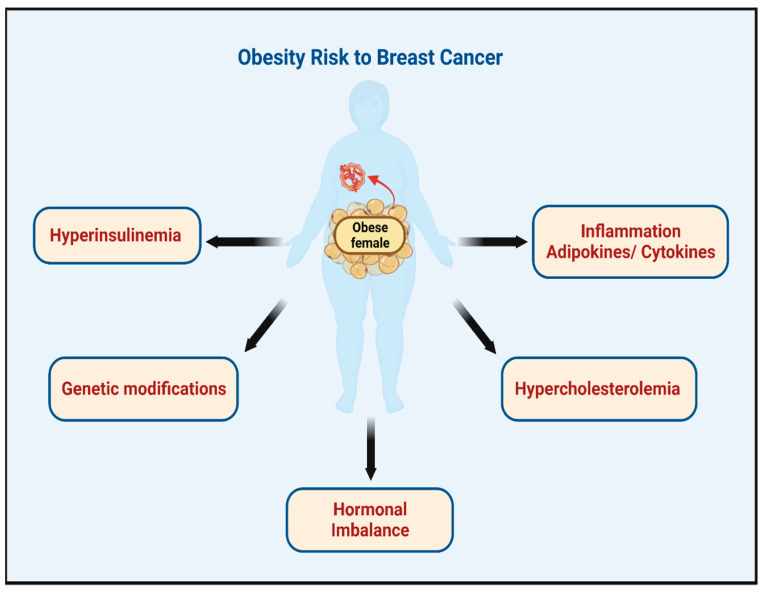
The main molecular changes induced by obesity and related to BC risk. Created with BioRender.com, Toronto, Canda.

The association between obesity and BC risk is believed to be because obesity elevates levels of hormones and hormone receptors including the estrogen receptor-positive BC with a relationship to triple negative and the human epidermal growth factor receptor-2 [18]. In addition, obesity can induce genetic mutation/epigenetic modification due to the presence of a high amount of fat tissues that causes a metabolic imbalance of circulating levels of insulin [29,30,31]. Furthermore, inflammatory cytokines can be induced, which can increase the chances of the development and/or progression of certain types of cancer, including BC [11,30,32]. Figure 1 highlights the main molecular changes of obesity and risk to BC.

**Figure 2:**
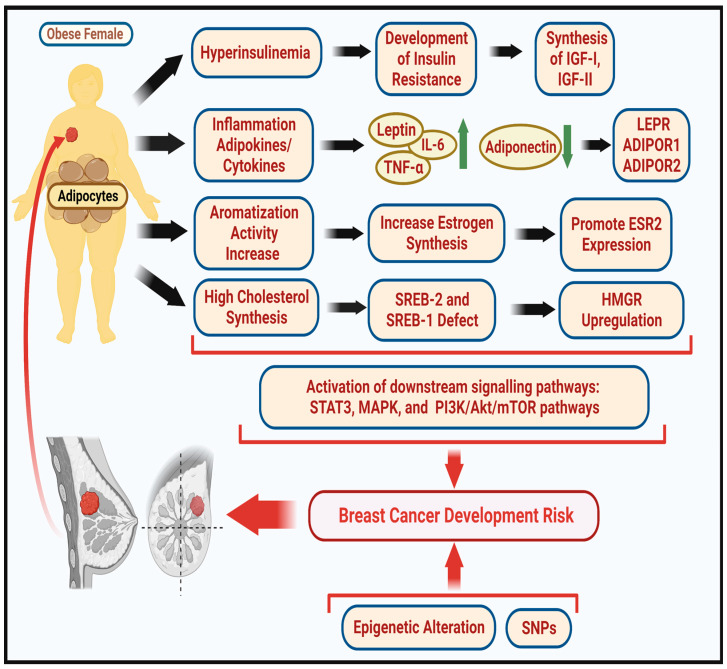
Molecular and genetic modifications in obesity risk to BC development. Molecular pathway changes in obesity, including increased insulin levels and development of insulin resistance, inducing the synthesis of insulin growth factors I and II (IGF-I, IGF-II), inflammation reactions, and their imbalanced levels of adipokines and cytokines and expression of leptin receptor LEPR and adiponectin receptor ADIPOR1-2. The high aromatization reactions in the fat tissues lead to the high production of estrogen hormone. High levels of cholesterol synthesis are regulated by transcription faction sterol regulated element binding protein-2 (SERP-2, SERP-1). Downstream activation of molecular changes such as Janus kinase/signal transducer and activator of transcription 3 (JAK/STAT3), mitogen-activated protein kinase (MAPK), and phosphatidylinositol 3-kinase/v-Akt murine thymoma viral oncogene homolog/mammalian target of rapamycin (PI3K/Akt/mTOR occur. The molecular changes induce epigenetic alterations and single nucleotide polymorphism (SNP) risk. All these biological modifications induce BC development. The illustration was created with BioRender.com, Toronto, Canda.

Different reports have described several molecular and genetic interrelations between body adiposity and BC [21], as summarized in Figure 2. However, these molecular relationships between obesity and BC risk remain incompletely understood [21]. Therefore, this review aims to discuss the different molecular mechanisms and genetic interactions in women with obesity that might be associated with BC risk and development.

## 2. The Distinction of Obesity to BC Risk among Pre- and Post-Menopause

As mentioned above, obesity is a multifactorial condition associated with multiple health complications. The incidence of obesity is growing, affecting more than 600 million adults globally, accounting for about 13% of the global population [33]. The impact of obesity on BC risk in women has recently received significant attention in many studies, especially since global BC risk and incidence have increased dramatically in the past decade [20,34]. Moreover, associations of weight gain changes with BC outcomes have been assessed during or after adjuvant therapy in various studies [35]. A study reported associations of weight gain after chemotherapy in patients with a younger age, such as premenopausal women, supporting that there is weight gain with chemotherapy [36].

BC is a heterogenous disease associated with various molecular and genetic alterations [34]. The classification of BC subtypes is based on the expression of the estrogen receptor (ER), progesterone receptor (PR), and human epidermal growth factor receptor-2 (HER2) (Table 1) [37,38]. In addition, genetic classifications including BRCA1 DNA repair associated (*BRCA1*), BRCA2 DNA repair associated (*BRCA2*), and tumor protein p53 (*TP53*) mutations can provide for more detailed description of the tumor’s molecular biology, which can improve disease prognosis [37,38].

Nevertheless, whether obesity affects or promotes tumorigenesis in all BC subtypes based on *ER*, *PR*, and *HER2* expression status it is not fully understood [39]. In addition, the relationship between obesity and BC in pre- and post-menopausal women is controversial (Table 2). The correlation between obesity and premenopausal BC risk varies among disease subtypes [34]. However, other studies found no association [34,35]. Moreover, several studies have demonstrated that obesity is associated with lower ER+ BC risk in premenopausal women [40,41,42]. The ER–BC association with obesity showed a higher risk for premenopausal and TNBC in most studies [24,41,43,44]. The Cancer and Steroid Hormone (CASH) case control study of 3432 women with BC found a strong positive association between BMI and premenopausal TNBC risk. However, some risk factors differ by molecular subtypes, suggesting BC heterogeneity among young age females [45]. The HER2^+^ BC subtypes were not significantly associated with BMI ≥ 25kg/m^2^ in premenopausal women [46]. Chen et al. reported that while patients with BC and overweight or obesity might have a high frequency of HER2^+^ subtypes, their risk is still not significantly elevated: odds ratio (OR) = 1.24 (95% confidence interval (CI) = 0.81–1.88) and OR = 1.41 (95% CI = 0.92–2.16) for women with overweight and obesity, respectively [47]. Many epidemiological studies have shown that BC risk is much greater for postmenopausal than premenopausal women with obesity [48]. After menopause, the adipose tissue mass will become the primary site of estrogen production, which will be much larger in women with obesity [49]. Epidemiological studies on all BC subtypes have shown that obesity contributes to worse disease-free survival [50]. Chen et al. showed that postmenopausal women with obesity had an overall relative risk of about 1.33 for developing ER^+^ BC [44]. In addition, other studies have reported that obesity could also be associated with TNBC incidence and progression in postmenopausal women [51,52]. However, the relationship between obesity and HER2^+^ BC remains incompletely understood. Some studies have found obesity associated with low survival in women with HER2^+^ BC [53,54]. Modi et al. investigated the association between obesity and HER2^+^ BC in a large high-quality dataset. While they found that higher BMI was independently associated with worse survival in women with early HER2^+^ BC (5099 patients), they unexpectedly found that higher BMI was independently associated with better survival in women with advanced HER2^+^ BC (3496 patients) [55].

The available studies on premenopausal BC and its association with obesity are generally limited. Most research on postmenopausal BC and its risk factors, including obesity, suggest that premenopausal BC may share the same risk factors [56]. In addition, increasing evidence indicates different risk factors for premenopausal BC, including dense breasts and a family history of BC [56]. Obesity risk factors appear to have a low effect on and contribution to premenopausal BC [57]. Therefore, most published evidence discusses the molecular and genetic associations of obesity with BC in postmenopausal women [57].

## 3. Adiposity and Adipokines Secretion

Obesity progresses due to high caloric intake [58]. The additional energy is stored as lipids in the adipose tissue and may accumulate in other metabolic organs (such as the liver) and skeletal muscle [58]. The enlargement of adipose tissue can significantly change the normal metabolic flow and induce transformation in cell metabolic signaling and response [58]. These metabolic alterations include increased cellular glucose uptake, growth, and proliferation, which can stimulate angiogenesis [59,60,61,62].

Adipose tissue can act as a secreting organ and is considered an active endocrine organ [63]. Adipose tissue comprises visceral adipose tissue and subcutaneous adipose tissue. The adipose tissue is the core organ for body energy homeostasis (i.e., endocrine dynamics) [32,63]. Therefore, triglycerides are the primary form of energy storage in adipose tissue [32]. Adipocytes secrete different cell types, including adipocytokines, preadipocytes, endothelial cells, and immune cells [64]. Adipocytes account for the bulk of cells in human breast tissues and about 10% of epithelial tissues [32]. Therefore, obesity is considered the expansion of the white adipose tissue, which releases high amounts of free fatty acids into circulation via the lipolysis mechanism, increasing their serum levels [65]. The accumulation of this lipid metabolic action cycle is considered the primary cause of insulin resistance and other metabolic dysfunction [64,65]. Consequently, fatty tissues promote pre-inflammatory and proto-oncogene development in individuals with obesity [66,67]. Recent epidemiological and translation studies have shown that local ectopic breast adipose tissue has deleterious and tumorigenic effects on BC development and progression [13,68,69]. Furthermore, obesity reflects increased fat tissue that produces small peptide hormones and growth factors called adipokines, which are involved in various metabolic and inflammatory functions including body weight balance, appetite regulation, glucose homeostasis, and blood pressure control [64].

In 1994, the first adipokine was identified and named leptin, a hormone secreted explicitly by the adipose tissue [64,70]. Other adipokines have since been identified, including adiponectin, tumor necrosis factor-alpha (TNF-α), and interleukin (IL)-6 [71,72]. Secretion of these adipokines has been associated with tumor growth [32,72]. Moreover, recent reports suggest strong associations between overweight/obesity and insulin resistance and adipokines in postmenopausal women with BC [67]. More than 10 adipokines have been associated with BC risk [66,73,74]. The examination of fat tissue in individuals with obesity showed that more adipocytes produce leptin [34,66]. Hypoxia alters gene expression in adipocytes, particularly of proinflammatory adipokines and immune factors [34]. In addition, the adipose tissue of individuals with obesity develops chronic inflammation, which is induced by nuclear factor kappa Β (NF-κΒ). Different studies have examined several adipokines in BC tissue, including leptin, adiponectin, TNF-α, and IL-6 [67,71,75]. Leptin and adiponectin levels showed considerable variation in BC tissue [67,71,75].

### 3.1. Leptin

Leptin is a 16 kDa polypeptide produced mainly by adipocytes in healthy and malignant tissues [76]. Many studies have shown that leptin is overexpressed in individuals with overweight and obesity [30,77,78,79]. In addition, leptin has been shown to have several roles in promoting normal and tumor cell growth and migration and angiogenesis [30,77,78,79]. Leptin was first discovered in 1994 by Friedman et al. [70,76]. Leptin is a product of the *LEP*/*OB* gene and exists in circulation in free and bound forms [70]. Leptin was found to be expressed in normal and tumor mammary epithelial cells [80,81]. Leptin mediates its effect via the leptin receptor (LEPR), a universally expressed transmembrane protein [70,76]. In addition, LEPR was found to be expressed in normal mammary epithelial and human BC cell lines [80,82]. Therefore, leptin secretion is proportional to the mass of the adipose tissue and reflects energy adequacy, leading to appetite suppression [30,79]. Giordano et al. showed that leptin was associated with mammary cell tumor development through cell-to-cell signaling via exosome biogenesis regulation and release of different BC cells, such as MCF-7 (ER^+^) and MDA-MB-231 (TNBC) cells [83].

Much research has shown that leptin directly stimulates cell proliferation via the LEPR, activating intracellular pathways, such as mitogen-activated protein kinase (MAPK), phosphatidylinositol 3-kinase/v-Akt murine thymoma viral oncogene homolog (PI3K/Akt), and Janus kinase/signal transducer and activator of transcription 3 (JAK/STAT3), and proteins, such as Jun N-terminal kinase (JNK), protein kinase C (PKC), p38 MAPKs, and NF-κB [30,80]. NF-κB regulates the transcription of various genes involved in cell proliferation, such as cyclin D1 (CCND1), MYC proto-oncogene bHLH transcription factor (c-MYC), Jun proto-oncogene AP-1 transcription factor subunit (JUN), Fos proto-oncogene AP-1 transcription factor subunit (FOS), and B-cell leukemia/lymphoma 2 (BCL2) [84]. Moreover, leptin was shown to directly activate ER signaling, promoting aromatase activity in BC cells [85]. Furthermore, leptin activated LEPR-expressing macrophages and stimulated the secretion of several proinflammatory cytokines, including IL-1, IL-6, IL-11, TNF-α, and nitric oxide, modulating macrophages [85]. Therefore, various studies found increased leptin and LEPR expression in primary and invasive ductal BC compared to non-BC tissues [85]. Jarde et al. studied the effect of leptin and LEPR expression in primary mammary tumor cells [86]. They found that leptin and LEPR were expressed with estrogen receptor expression, which all interact to promote BC progression. Moreover, they found that LEPR contributes to increase tumor size [86]. Another study by Yan Wang et al. found that leptin and LEPR expression were significantly correlated with lymph node metastasis and Ki-67 expression, respectively [87]. They concluded that high leptin and LEPR expression were risk factors for BC development [87]. Consequently, several studies support the theory that high serum leptin levels correlate with BC development and progression [88,89].

### 3.2. Adiponectin

Adiponectin is a polypeptide composed of 244 amino acids (about 30 kDa) and is considered a complement-related hormone secreted by adipocytes [90,91]. Adiponectin exerts its function through two receptors: adiponectin receptor 1 (ADIPOR1; 40 kDa), and 2 (ADIPOR2; 35 kDa). These receptors are widely expressed in several tissues, including breast tissue, skeletal muscle, and liver [92,93]. Adiponectin receptors are expressed in normal and cancerous tissues [93]. Since circulating adiponectin levels are inversely proportional to adipose tissue mass, they have been shown to protect against the development of other obesity-related disorders, including metabolic syndrome, diabetes, cardiovascular disease, and cancers [93]. In addition, different studies have found that adiponectin exerts anti-proliferative, anti-migratory, and pro-apoptotic effects [94,95]. Moreover, another study found an inverse association between adiponectin levels and carcinogenesis [93]. Therefore, adiponectin activates the AMP-activated protein kinase (AMPK)/serine/threonine kinase 11 (STK11/LKB1) pathway, which is involved in regulating cellular metabolism, proliferation, apoptosis, and angiogenesis [93]. Adiponectin exerts its effect by binding to its receptor, causing translocation of the STE20-related adaptor alpha (STRADA/STRAD) protein from the cell nucleus into the cytoplasm, leading to the phosphorylation of LKB1. This process activates the AMPK pathway, which inactivates the PI3K/Akt, NF-κB, and JAK2/STAT3 pathways [96,97]. Brakenhielm et al. investigated whether treating physiologic adiponectin levels could inhibit mouse fibrosarcoma tumor neovascularization [32]. Therefore, low serum adiponectin levels in individuals with obesity could indicate a high risk of developing cancer [32]. However, the direct effect of adiponectin on breast epithelial cell growth, proliferation, and differentiation still needs further research [76,98]. Studies reported that ligand binding to ADIPOR1 and ADIPOR2 could activate the peroxisome proliferator-activated receptor (PPAR)-α pathway. PPAR-α activates the transcription of diverse genes involved in different processes, including cell proliferation and differentiation. Interestingly other studies showed that treatment with PPAR-α agonists could help improve insulin resistance [90].

### 3.3. TNF-α

TNF-α is expressed in white adipose tissue and was first identified in rodents, with markedly increased levels in obese models [99]. It has been suggested that TNF-α might be involved in insulin resistance development via different mechanisms, such as suppression of insulin receptor signaling [100]. While TNF-α levels released by adipose tissues are unknown, the association between obesity and TNF-α and its receptor mechanism is well explained [101]. TNF-α induces various autocrine and paracrine effects in the adipose tissue, including apoptosis and the synthesis of cytokines and adipokines [102]. In addition, Nascimento et al. found that TNF-α plays a role in regulating IL-6 synthesis [103,104]. Moreover, TNF-α stimulated estrogen by increasing aromatase expression in adipose tissue [105]. Therefore, obesity increases circulating TNF-α levels, increasing the risk of breast tumorigenesis related to insulin resistance and IL-6 synthesis.

### 3.4. IL-6

IL-6 is a small protein type of cytokine expressed and secreted by adipocytes [55]. IL-6 plays an important role in BC progression by stimulating its downstream effector signaling pathways [106]. STAT3 is one downstream effector pathway for IL-6, and it is highly active in >50% of BCs, suggesting a cancer-promoting effect [106,107]. Various studies have reported that STAT3 induces crosstalk between the JAK/STAT pathway and other signaling pathways, such as MAPK/MEK/ERK and PI3K/Akt, promoting cancer progression, chemo-resistance development, and the epithelial–mesenchymal transition [108,109]. IL-6 was shown to enhance cell migration by activating the MAPK pathway and inhibiting the activity of proteases involved in apoapsis. Therefore, IL-6 is considered an antiapoptotic factor [110].

IL-6 is highly expressed in obesity, and elevated IL-6 levels in circulation are related to insulin resistance [111,112]. Therefore, high serum IL-6 levels are associated with worse prognosis and survival, causing low responses to chemotherapy and endocrine therapy [113,114]. Moreover, IL-6 stimulates osteoclast synthesis and prevents dendritic cell differentiation, inducing metastatic growth [115]. Additionally, IL-6 stimulates aromatase expression in adipose tissue in vitro and in vivo, activating estrogen biosynthesis and correlating with BC development [105].

## 4. The Hormonal Imbalance Effect

The association between obesity and BC risk in postmenopausal females is commonly related to hormone imbalances, such as estrogen imbalances [116,117,118]. Therefore, estrogen levels increase with the adipose tissue mass in postmenopausal females, especially those with obesity, due to increased aromatase activity in estrogen biosynthesis [17,32,117,118]. While the ovaries are the primary source of estrogen in premenopausal women, estrogen is also synthesized in the adipose tissue [17,19], indicating that aromatization activity also exists in adipose tissues [17,19].

Therefore, high aromatization activity in adipose tissues can lead to increased gene expression of different cytokines and insulin resistance development [32,117,118]. Consequently, various obesity research data show the extreme production of estrogen in adipose tissue, which is related to the effects of adipokines and inflammatory cytokines [117,118]. In addition, these molecular actions lead to excessive insulin-like growth factor (IGF) pathway activity, which is mediated in BC cells by molecular signaling through other mechanisms and estrogen receptor 2 (ESR2) pathways [119,120]. Therefore, estrogen is an independent factor that binds to the ERs expressed by BC cells [22]. Consequently, >70% of BC cases express ESR2α, determining patients’ prognoses [121]. Other factors, including high TNF-α and IL-6 secretion levels by adipose tissues, act as autocrine or paracrine mechanisms that promote aromatase production [105,122].

Based on previous evidence, estrogen is known to be crucial for normal mammary development and ductal growth and has an essential role in human BC progression and development [4,22,118]. Therefore, long-term exposure to estrogen or/and progesterone can increase the *ESR2* expression in mammary epithelial cells, increasing BC risk [4,22,118].

## 5. Hyperinsulinemia and the Insulin Resistance Effect

Obesity development has been associated with high insulin levels, leading to hyperinsulinemia [32]. The long-term exposure of body tissues to high circulating insulin levels induced certain metabolic modifications in adipose tissue, causing various health problems, including risks of metabolic syndrome and diabetes [123]. Insulin resistance develops as a metabolic adaptation in which high levels of non-esterified fatty acids are related into the circulation by adipose tissues, mainly intrabdominal adipose tissue. Therefore, the high fatty acid levels released from adipose tissues direct the liver, muscle, and other tissues to stimulate adipose tissues, inducing fatty acid storage and oxidation [21,124]. This process initiates several physiological changes strongly associated with insulin resistance development and inflammation with abnormal adipokines secretion [21,124]. Consequently, the high circulating glucose levels induce an increase in insulin production from the pancreas in fed and fasting states, which may cause type 2 diabetes mellitus [32]. The high levels of insulin secretion have a direct signaling effect, activating the tyrosine signaling cascade [32]. This molecular action has been associated with the risk of BC development [32]. Previous animal studies have reported this particular molecular response, indicating that diabetic animal models can increase the induction of mammary tumors [125,126]. Moreover, the impact of hyperinsulinemia can increase the formation of IGF-1 synthesis [32,127]. Different studies have reported the contribution of IGF-1 in BC [32]. The IGF family comprises IGF-1 and IGF-2 that bind to their own membrane-bound tyrosine receptor (IGF-1R and IGF-2R, respectively) and six IGF-binding proteins (IGFBPs) [7,8,9,10,11,12,13,14,15,16,17,18,19,20,21,22,23,24,25,26,27,28,29,30,31,32].

IGF-1 and IGF-2 generally function in an endocrine, paracrine, or autocrine manner for regulating cell growth, survival, and differentiation and can integrate with other growth factors, resulting in mitogenic and antiapoptotic effects [128,129,130]. IGF-1 can target receptors on human breast epithelial cells to stimulate mitogenic and antiapoptotic effects [32]. IGF-1 binding to its receptor IGF-1R activates tyrosine kinase activity, phosphorylation reactions, and different intracellular signaling pathways, including the PI3K and MAPK signaling pathways [32]. A previous study showed that the PI3K and MAPK pathways are crucial for IGF-1-stimulated proliferation of MCF-7 human BC cells in vitro [131]. In addition, *IGF-1* overexpression was particularly efficient in promoting tumor growth [32]. Moreover, around 50% of primary breast tumors overexpress *IGF-1R* compared with normal tissue, indicating that these carcinomas may have enhanced responses to the mitogenic and antiapoptotic effects of IGF-1 [119]. These findings are also supported by various studies on mouse models of BC that have shown the effect of hyperinsulinemia in stimulating BC proliferation and metastasis and greatly changing the response of tumors to PI3K inhibitors [119,130].

Therefore, previous studies suggest an association between IGF-1 and IGF-1R and BC risk in women with obesity. Moreover, the specific relationship between IGF-1′s mechanism and estrogen-mediated pathways was greatly enhanced in postmenopausal women with obesity [4,104].

## 6. The PI3K/Akt/mTOR Signaling Pathway Interrelation Effect

One major cellular signaling pathway stimulated in women with obesity is the PI3K/Akt/mammalian target of rapamycin (mTOR) pathway. mTOR is a serine/threonine protein kinase related to the PI3K family that interacts with various protein complexes, such as mTOR complex 1 (mTORC1) and 2 (mTORC2) [132]. Activating the mTOR pathway mediated various cancer features, including angiogenesis and cell proliferation [133,134]. This pathway is activated by amino acids and IGFs stimulated in adipocytes by phosphorylation [120,135].

Around 70–75% of BC cases express *ESR2*, implying estrogen-dependent tumorigenesis [136]. Therefore, 17β-estradiol activation by ESR2-α is crucial for inducing cancer cell proliferation and preventing apoptosis [137]. Moreover, various studies have shown that PI3K/Akt/mTOR pathway activation affects the growth rate of ESR2^+^ BC [136].

In adipose tissues, mTOR-mediated phosphorylation at Ser501/503 modifies the binding of the adaptor protein growth factor receptor bound protein 10 (GRB10) to the insulin receptor and regulatory-associated protein of mTOR complex 1 (RPTOR) [121]. However, the dissociation of RPTOR from mTOR downregulates mTORC1 signaling [138,139]. In addition, obesity-induced insulin resistance in BC relies on chronic activation of mTORC1 [121]. *RPTOR* mRNA levels were found to be higher in tumor compared to normal tissues [136,139]. Furthermore, *RPTOR* expression was associated with a higher tumor grade [140]. Following estrogen stimulation, ESR2α binds to RPTOR and induces its translocation into the nucleus [140]. Consequently, the effect of estrogen in guiding the interaction between mTORC1 and ESR2α, in addition to RPTOR translocation into the nucleus, mediates the phosphorylation of ESR2α on Ser104/106 [121,141].

Therefore, the interaction between ESR2α and PI3K/Akt/mTORC1 signaling confirms the strong activation of oncogenic signaling in ESR2α^+^ BC cells [121,141]. Consequently, many studies have shown the potential of mTOR as a therapeutic target in treating BC [140]. Cheng et al. demonstrated a positive association between body fatness in women with high BMI and mTOR pathway activation, indicating phosphorylated mTOR expression in BC [138]. Several clinical trials have investigated using a combined therapeutic strategy against mTOR [140]. Consequently, these findings suggest that mTOR inhibition should be considered a treatment approach to prevent BC recurrence in women with obesity [138].

## 7. Enhancement of the Cholesterol Synthesis Effect

Several studies have identified cholesterol as one of the general risk factors for BC [142]. Both obesity and diets high in saturated fats are associated with dyslipidemia and hypercholesterolemia [143]. Cholesterol is the principal structural component of cell membranes and is required for cancer cell proliferation [144]. In addition, cholesterol is the primary precursor for steroid hormone synthesis, including estrogen and progesterone, which potentially induce BC development [143]. Much research has indicated that hypercholesterolemia is an independent risk factor for BC in postmenopausal women [117,145].

The biochemical synthesis of cholesterol in tissues occurs through the mevalonic acid pathway, a series of enzymatic reactions starting with the rate-limiting step 3-hydroxy-3-methylglutaryl-CoA reductase (HMGCR) [146]. *HMGCR* expression is regulated by the transcription factor sterol-regulated element binding-protein-2 (SREBP2) [142]. SREBP2 belongs to the SREBP family of transcription factors, which includes other isoforms such as SERBP1 [147,148]. These transcription factors help regulate genes involved in lipid synthesis and uptake pathways, including cholesterol [149]. Several clinical studies have demonstrated a molecular association between high cholesterol levels and BC pathogenesis. Additionally, clinical studies have shown that BC patients treated with statins had low BC recurrence [150,151,152,153]. Statins therapy inhibits HMGCR, inhibiting the synthesis of cholesterol and its metabolites, including 27-hydroxycholesterol [153]. The 27-hydroxycholesterol metabolite has been shown to regulate BC metastasis by restoring the tumor microenvironment and promoting resistance to ferroptosis, a crucial feature in metastatic cancer cells [154]. Moreover, cholesterol was shown to increase the stemness of the cancer cells that initiate cancer metastasis [155]. Furthermore, many studies have shown that enhanced cholesterol synthesis can stimulate oncogenic signals, leading to cancer cell growth and proliferation, specifically by activating the PI3K/Akt/mTOR and AMPK signaling pathways [156]. Additionally, cholesterol binding to specific cancer-related proteins can induce changes in their structure or activity, potentially further contributing to BC development and progression [143,157].

Various studies have reported several molecular/cellular defects that can occur in cholesterol metabolism. The SREBP2 transcription factor regulates cholesterol homeostasis and uptake via the liver X receptors (LXRs) [144]. The LXRs are members of the nuclear receptor family of ligand-regulated transcription factors [158]. Therefore, previous research has suggested that SREBP2 may be involved in BC progression by upregulating the expression of genes responsible for cholesterol synthesis, such as HMGCR, and import [142,146]. One study demonstrated that *SREBP2* expression was significantly higher in BC than normal tissues and that SREBP2 may be involved in BC cell proliferation and migration. Additionally, SREBP2 was also associated with a higher risk of BC relapse and recurrence in patients. Moreover, SREBP1 was found to play a role in the metabolic reprogramming and upregulation of BC cells, leading to the increased production of cholesterol, fatty acid, and triglyceride metabolism-related genes [159]. This SREBP1-mediated upregulation in BC cells can eventually exacerbate the metabolic dysregulation that drives BC progression [160]. However, further studies are needed to investigate SREBP1 regulation and the role of SREBP2 in providing potential therapeutic targets for BC.

## 8. The Genetic Interactions of Obesity with BC Risk

### 8.1. Epigenetics in Obesity and BC Risk

Recent studies have suggested that obesity-induced epigenetic variations increase the risk of developing BC [161]. Epigenetic changes are genomic alterations that affect gene expression without altering the underlying DNA sequence [162]. These epigenetic alterations, including DNA methylation and histone modifications, can all impact gene expression through the loss of tumor suppressor genes and the aberrant expression of oncogenes, thereby leading to cancer growth and progression [161,163]. Studies have also suggested that epigenetic changes can influence hormone responses, possibly by altering the activity of hormone receptors, thereby playing a role in BC risk [163].

Studies on women with obesity have found higher levels of these epigenetic modifications, potentially increasing their BC risk [164]. Furthermore, epigenetic changes may lead to a dysfunctional immune response that cannot recognize and remove BC cells [164]. A high-fat diet is associated with higher BMI, and diets rich in saturated fats have been shown to affect DNA methylation. Perfilyev et al. investigated the effect of seven weeks of high saturated fat intake on genome-wide DNA methylation in the subcutaneous adipose tissue in young, healthy humans [165]. They observed a modifying effect on DNA methylation for 125 genes, including adiponectin, CQ1, and collagen domain-containing (ADIPOQ); all were methylated in adipose tissue [29,165].

Studies on the impact of obesity on gene-specific DNA methylation and BC have found different associations, albeit not differentiated by menopausal status. Therefore, several studies demonstrated an association with *BRCA1* hypermethylation in women with obesity [166]. However, obesity was not associated with *BRCA1* or *BRCA2* promoter hypermethylation in a healthy cohort of mostly female nurses with obesity aged 40–60 years [166].

Regarding global DNA methylation in cancer, the CpG methylation forms are considered global hypomethylation, which is gene-specific hypermethylation [163]. Therefore, global hypomethylation related to cancer development and progression occurs through oncogene activation and chromosomal abnormalities [167,168]. Alterations of global CpG methylation can be measured via repetitive elements in the DNA (e.g., LINE-1 and Alu), the percentage of methylated DNA with the luminometric methylation assay (LUMA), or the 5-methyldeoxycytidine level based on the mean methylation intensities of Infinium HumanMethylation450 probes (β-value) [73,169]. Severi et al. reported an inverse association between BC and global CpG methylation in peripheral blood BC using the LUMA approach [170].

A growing body of recent evidence demonstrates the modulation effect of obesity on BC methylation. A population-based study by McCullough et al. on postmenopausal patients with BC investigated global DNA methylation in white blood cells (WBCs), finding that women without obesity in the highest LUMA score quartile had an increased BC risk [51]. However, no association was found in patients with obesity [51]. Moreover, patients with BC and high BMI showed more frequent hypermethylation of the Ras-association domain family member 1 isoform A (*RASSF1A*) and *BRCA1* genes (120 surgically excised tumors) [171]. The Long Island BC Study Project found hypermethylation of the hairpin-induced 1 (*HIN1*) gene in >500 postmenopausal BC tumors [171]. The *HIN1* gene encodes a protein with a significant role in cell growth and invasion [172].

Furthermore, BC-specific mortality was higher in patients with obesity and WBCs with low LUMA levels and hypermethylation of the adenomatous polyposis coli (*APC*) and twist family bHLH transcription factor 1 (*TWIST1*) genes [173]. Decreased LUMA levels in WBCs was also correlated with high mortality in patients with obesity. The Carolina BC study demonstrated global CpG methylation in 345 BC tumors using an Illumina-based approach, showing that 87% of the probes had increased β-values in patients with a BMI ≥ 30 kg/m^2^ [174]. Additionally, 21 gene loci were differentially methylated in patients with obesity with and without ESR1-positivity, which were reportedly involved in the immune response, cell growth, and DNA repair. Interestingly, DNA methyltransferase 3 beta (DNMT3B) showed the most significant difference in β-values between patients with and without obesity [174]. However, a meta-analysis found an association between higher physical activity and lower BC risk, suggesting a positive association between global CpG methylation and physical activity [175].

### 8.2. SNPs Associated with Obesity and BC Risk

Several studies have identified genetic associations between single nucleotide polymorphisms (SNPs) and obesity and BC risk, implicating elevated insulin levels, impaired glucose metabolism, and dyslipidemia in BC risk [176]. Furthermore, some of these SNPs are known to alter the expression of genes involved in DNA repair and synthesis and may increase the risk of genetic mutations [29]. Additionally, these SNPs have been associated with changes in hormone receptor expression and signaling pathways, which may be involved in promoting tumor growth and progression [29].

Recent genome-wide association studies have identified several SNPs associated with high BMI and BC (Table 3). The FTO alpha-ketoglutarate-dependent dioxygenase (*FTO*) gene was reported to be associated with obesity [177]. FTO was found to be involved in appetite and food intake [178,179]. The *FTO* gene is located on chromosome 16q12.2 and is expressed in all tissues, with higher expression levels in the liver, brain, hypothalamus, and visceral fat [180]. Several *FTO* SNPs have been associated with cancer, including rs8050136, rs9939609, rs1477196, rs1121980, rs6499640, rs17817449, rs8047395, rs7206790, and rs11075995 [181,182]. The *FTO* SNP rs9939609 was reported to have AA genotype frequencies of 12% and 26% in male and female subjects, respectively [183]. The *FTO* SNP rs9939609 was correlated with BC and the effect status of ERs and the PI3K/Akt signaling pathway [31]. A case-control study by Doaei et al. found a significant positive association between BC and dietary fat intake in women with the risk allele of *FTO* SNP rs9939609 [177].

Several studies on *ADIPOQ* gene polymorphism found that homozygous carriers of the BC risk allele (T) for SNP rs1501299 had higher adiponectin levels than those with GG or TG genotypes [176,184]. Additionally, the TG and GG genotypes for SNP rs2241766 were associated with increased serum adiponectin levels and decreased BC risk [29,184]. Therefore, SNPs that induce low adiponectin levels correlate with high BC risk, which is proportional to adiposity [184]. Therefore, low serum adiponectin levels underlie the high BC risk of women with obesity [176,184].

Moreover, the leptin (*LEP*) gene SNP −2548G/A has been associated with BC risk [185]. Therefore, leptin is involved in body weight homeostasis [185]. Decreased leptin levels were associated with BC risk in women with obesity [185].

Other genes include the proto-oncogene FER tyrosine kinase (*FER*). A recent study on *FER* SNP rs10447248 (T/C) reported that postmenopausal women with a BMI ≥ 30 kg/m^2^ had a ~2-fold higher BC risk when homozygous for the minor T allele than the major C allele with both model 1 (hazard ratio (HR) = 2.20, 95% CI = 1.08–4.49) and model 2 (HR = 2.53, 95% CI = 1.17–5.45) [186]. Therefore, FER induces NF-κB activation and IL-6 signals to regulate STAT3 phosphorylation [187,188]. This action connects adiponectin and obesity to BC risk [186,187,188].

## 9. Conclusions

BC development is indirectly associated with obesity in women, especially those in their postmenopausal phase. Obesity is a complex health condition that involves different molecular changes affecting cellular metabolism and DNA, which can lead to tumor growth. The major pathological issue of obesity begins with the development of chronic inflammation, which affects body immunity and leads to cancer initiation. Therefore, the inflammation process induces a series of downstream effector signaling pathways that promote cancer development and proliferation. In addition, these actions involve high estrogen levels due to greater fat mass, which increases aromatase activity. The epigenetic and genetic modifications caused by the various molecular effects of obesity are all associated with BC progression. Altogether, these molecular alterations contribute to BC development. Further investigations are required to delineate further the molecular relationship between obesity and BC risk. In addition, investigating these mechanisms and genetic background is important for developing new preventative and therapeutic strategies targeting BC, thereby improving BC diagnosis and prognosis.

## Figures and Tables

**Table 1:** Classification of BC subtypes based on hormone receptors. Key: ER, estragon receptor; PR, progesterone receptor; HER2, human epidermal growth factor 2; TNBC, triple-negative BC.

BC Subtypes	ER	PR	HER	Prognosis	Frequency (%)
**Luminal A**	Yes	Yes	No	Good	50
**Luminal B**	Yes	Some cases	No	Moderate	15
**HER2**	Some cases	Some cases	Yes	Moderate/Poor	20
**TNBC**	No	No	No	Poor	15–25

**Table 2:** BC receptor subtypes and their association with obesity in pre- and post-menopausal women. ER^+^/PR^+^, estragon receptor positive and progesterone receptor positive; TNBC, triple-negative BC; HER2^+^, human epidermal growth factor 2 positive.

Phase	BC Receptor Subtypes	Obesity Association with BC Outcomes	Study
**Premenopausal**	ER^+^/PR^+^	- Obesity was associated with lower ER+ BC risk before menopause. - There was an inverse association between BMI and ER+ BC risk before menopause.	[34,36,38]
	TNBC	- Obesity was associated with a higher risk of premenopausal ER^−^ BC and TNBC in most studies. - Two meta-analyses of 620 women and women with TNBC reported an 80% and 43% higher risk of developing TNBC with obesity.	[34,35,37,51,52,53]
	HER2^+^	- Nonsignificant association or increased risk of BC.	[41,42]
**Postmenopausal**	ER^+^/PR^+^	- Increased risks of developing ER^+^ BC.	[39]
	TNBC	- Obesity was associated with postmenopausal TNBC incidence and progression.	[44]
	HER2^+^	- Obesity was consistently associated with worse overall survival in patients with early HER2^+^ BC.	[46,47]

**Table 3:** Summary of reported obesity SNPs associated with BC risk.

Gene	Obesity SNP Associated with BC	SNP’s Molecular Effect	Reference
*FTO*	rs9939609	Affects the status of ERs and PI3K/Akt signaling pathway.	[175,180]
*LEP*	−2548G/A	Associated with higher leptin levels.	[184]
*ADIPOQ*	rs2241766 rs1501299	Induces low adiponectin levels inversely proportional to adiposity.	[163,183]
*ADIPOR1*	rs7539542 rs2232853	Alters mRNA levels of the receptor to modulate ADIPOR1 mRNA levels.	[183]
*FER*	rs10447248	Increases NF-κB activation and IL-6 signals to regulate STAT3 phosphorylation associated with BC risk through adiponectin and obesity.	[185,186,187]

## Data Availability

Not applicable.
